# Mr. Jeaffreson’s Operation for Ovarian Dropsy

**Published:** 1844-02-24

**Authors:** Wm. Jeaffreson

**Affiliations:** Framlingham


MR. JEAFFRESON'S OPERATION FOR OVARIAN DROPSY.

To the Editor of the London Lancet.

Sir,—Dr. Clay, of Manchester, having politely sent me a report of his seventh successful *major operation* for extripation of diseased ovary, without noticing my remarks, written privately to him soon after his first cases were printed, I beg, as the *originator* of the *minor operation*, for space in your Journal to enable me to state fairly, to the profession, the question at issue between us: namely, whether it be better to lay open the cavity of the abdomen, from the ensiform cartilage to the pubis, under the *apprehension* that adhesion *may* exist between the diseased structures and surrounding parts; or first, to perform the minor incision, and, should that be found insufficient, to enlarge the wound to the requisite extent?

As Dr. Clay acknowledges the impossibility of judging previously to incision, whether or not adhesions exist, I shall conclude by stating that in my first successful operation, published in the "Transactions" of the Provincial Medical Association, the disease had continued, during several years, without forming adhesions. This woman continues in good health, having since been pregnant four times, and given birth to four healthful children. I have since witnessed another successful *minor* operation for the cure of ovarian dropsy, which had progressed more than twenty years, during which period tapping had been thrice repeated. In that case, also, there was no adhesions. The lady has since enjoyed, during several years, and still enjoys, good health. I am, Sir, yours respectfully,

WM JEAFFRESON.

*Framlingham*, Nov. 6, 1843.

